# Integrated Analysis of Behavioral and Physiological Effects of Nano-Sized Carboxylated Polystyrene Particles on *Daphnia magna* Neonates and Adults: A Video Tracking-Based Improvement of Acute Toxicity Assay

**DOI:** 10.3390/bios16010010

**Published:** 2025-12-23

**Authors:** Silvia Rizzato, Antonella Giacovelli, Gregorio Polo, Fausto Sirsi, Anna Grazia Monteduro, Gayatri Udayan, Muhammad Ahsan Ejaz, Giuseppe Maruccio, Maria Giulia Lionetto

**Affiliations:** 1 Omnics Research Group, Department of Mathematics and Physics, CNR Nanotec-Institute of Nanotechnology, INFN Sezione di Lecce, University of Salento, Via Monteroni, 73100 Lecce, Italy; antonella.giacovelli@unisalento.it (A.G.); fausto.sirsi@unisalento.it (F.S.); annagrazia.monteduro@unisalento.it (A.G.M.); giuseppe.maruccio@unisalento.it (G.M.); 2 Department of Biological and Environmental Sciences and Technologies, University of Salento, Via Monteroni, 73100 Lecce, Italy; gregorio.polo@unisalento.it (G.P.); gayatri.udayan@unisalento.it (G.U.); muhammadahsan.ejaz@studenti.unisalento.it (M.A.E.); 3 National Biodiversity Future Center (NBFC), 90133 Palermo, Italy

**Keywords:** nanoplastics, environmental monitoring, ecotoxicity, miniaturized platform, *Daphnia magna*, biosensors

## Abstract

Nanoplastics pose significant environmental and public health risks, prompting the need for sensitive, cost-effective, and rapid assays for ecotoxicity assessment. The present work proposes the use of a portable smartphone-based platform to enhance traditional *Daphnia magna* acute toxicity assays by integrating behavior analysis and heart rate measurements. The aim is to improve sensitivity in detecting toxic effects of nanoplastics. In particular, the study focused on nano-sized carboxylated polystyrene (PS) nanoparticles. Two variability factors that could influence biological effects of nanoplastics, the particle size and the age of the organisms, were considered. Results demonstrated that the application of the proposed integrated approach allowed the detection of early subtle effects such as a significant impact on the heart rate and behavior of *Daphnia magna* under short-term exposure to PS carboxylated nanoparticles. In particular, a stimulation of heart rate was observed for both neonates and adults either for 40 nm or 200 nm particles after 48 h exposure, presumably attributable to an interference of carboxylated PS NPs with adrenergic-type receptors. Behavioral alterations were detectable for 40 nm particles but not for 200 nm ones consisting of a decrease in velocity and alterations of trajectories. Obtained results demonstrated the suitability of the proposed smartphone platform for friendly and real-time integration of behavioral analysis with physiological outcome measurements during acute exposure of *Daphnia magna* to nano-sized carboxylated PS NPs, expanding the sensitivity of the traditional acute toxicity tests. It offers a novel, cost-effective, and field-applicable method for environmental monitoring of nanoparticle toxicity and impact.

## 1. Introduction

Plastics and their derivatives find a great number of applications in various fields from daily life to industry and agriculture. However, their intensive use has increased the plastic contamination of natural environments, which has become a major environmental concern at the global level. It has been estimated that millions of plastic fragments enter into aquatic environments due to inadequate disposal, insufficient waste management, and urban run-offs [1].

Polystyrene (PS), a product of polymerization of styrene monomers, is one of the most extensively used types of plastic [2]. The number of polystyrene applications has grown in the last two decades. It is hardly biodegradable, and this facilitates its use in food and medical products and industrial devices. Although polystyrene can be recycled, only a small portion of produced polystyrene is actually recycled [3]. Non-recycled polystyrene disposables generate a problem of long-lasting environmental pollution. It is known that mechanical and photo-oxidative degradation of plastic materials in the environment produces microplastics (with diameters less than 5 mm), which, in turn, are degraded to nanoplastics (NPs) (with diameters of less than 100 nm) under the influence of biological, physical, and chemical factors [4]. For example, degradation of daily-use PS objects has been experimentally demonstrated to produce micro- and nano-PS particles [5,6]. These studies have demonstrated that microplastics and nanoplastics formed in the environment vary in size, particularly as a result of the degradation of foamed PS. In addition, primary micro- and nanoplastics, directly produced by the industry, are also released into the environment [2]. In the natural environment, NPs can undergo changes in their size, shape, charge, surface coating, and other properties which can affect their biological fate, mobility, bioavailability, and toxicity.

Both micro- and nanoplastics of various sizes have been detected mostly in the aquatic environment, where they are able to interact with biological systems [7,8], penetrate living organisms, and accumulate in the food chain [2]. They are able to exert toxic effects depending on concentration, particle size, exposure time, particle condition, shape, and polymer type. It is also known that the the surface chemistry of specific particles influences the interaction of the particles with biomolecules and this, in turn, influences their toxicity. The surface of polystyrene is commonly modified through a variety of chemical processes; some of them are carried out intentionally during the industrial production of plastics to improve the product characteristics for specific applications, while others occur during secondary microplastic fragmentation in the environment by oxidation and surface breakdown [2]. A common modification of PS is carboxylation, which consists in the addition of carboxyl groups to the surface of the polystyrene particles, resulting in a net negatively charged particle surface at a neutral pH.

In general, it is known that a positive surface charge of nanoparticles can increase cellular uptake and, in turn, toxicity due to binding to the negatively charged surface of the cell membrane. On the other hand, the presence of a negatively charged surface of a particle prevents endocytosis of the particle itself due to repulsive interactions with the cell membrane [9]. However, carboxylated PS-NPs with sizes ranging from 40 to 50 nm have been demonstrated to enter cells irreversibly in the A549 cell line [10].

Numerous studies have demonstrated several effects of nanoplastics on aquatic organisms, including the alteration of feeding, growth, and reproduction and impairment of immune response [11,12]. The significant environmental and health risks posed by micro- and nanoplastics have created an urgent need for sensitive, user-friendly assays that improve ecotoxicity assessment through rapid, cost-effective, effect-based detection.

Although most of the studies available in the literature have focused on the marine environment, recent studies have also documented micro- and nanoplastic contamination in freshwater environments [13,14] and their effects on freshwater organisms [15]. However, the number of works on the ecotoxicological effects of micro- and nanoplastics in freshwater organisms is comparatively far less that marine organisms.

The zooplankter *Daphnia magna* is a keystone species in freshwater food webs and a valuable organism in biosensing, particularly for environmental monitoring and toxicity assessment. It is widely recognized as a model organism in ecotoxicology, showing high sensitivity to chemical contaminants, wide geographical distribution, genetic homogeneity, a high reproduction rate, small body size, non-selecting filter feeding, and easy breeding in the laboratory. Zooplankters are known to ingest micro- and nanoplastics [16]. They are primary consumers in the food chain, allowing micro- and nanoplastics to enter the trophic chain.

Recent studies have focused attention on the toxic effect of nanoplastics, particularly polystyrene NPs, on this freshwater organism. Polystyrene NPs, even at environmentally relevant concentrations, can significantly alter growth, reproduction, and molting in *Daphnia magna*, raising concerns about their ecological impact on aquatic invertebrates [17,18,19,20]. Liu et al. studied the effects of 75 nm PS NPs at concentrations ranging from 10 to 400 mg/L on survival and expression levels of stress defense genes in *Daphnia pulex* in different age groups, highlighting the role of the age of the animal as a relevant factor for investigating toxic effects [21]. Lin et al. [22] found that acute toxicity of PS NPs to *Daphnia magna*, in terms of induced ROS production, MAPKs activation, and behavioral changes, was influenced by the functional groups present on the particle surface.

Several environmental guidelines use *Daphnia* as a standardized test species for ecotoxicity assessment of pollutants [23]. Immobilization of neonate *Daphnia magna* as a surrogate measure of their mortality following exposure to a chemical for 24–48 h is the basis of the internationally utilized OECD acute toxicity test 202 [24]. It is a widely used test for the assessment of toxicity of a wide array of pollutants including micro- and nanoplastics. Although mortality and immobilization are traditional endpoints, more sensitive responses are required for the determination of sub-lethal acute effects of toxicants, particularly emerging pollutants like micro- and nanoplastics. Indeed, mortality and immobilization are often less sensitive compared to physiological and behavioral responses, which can occur at lower concentrations of toxicants and provide early indicators of sub-lethal effects [25,26] that are often underestimated by standard approaches. Therefore, the development of more sensitive and user-friendly assays using *Daphnia* is crucial for improving ecotoxicity assessments of micro- and nanoplastics. The integration of these assays into routine testing can enhance the detection of subtle toxic effects and improve the relevance of toxicity assessments.

In this context, we recently optimized sensors for the detection of nano-sized PS NPs [27,28] and also reported effect-based analysis with applications in the field of environmental monitoring [29]. The aim of the present work was to develop a portable smartphone-based platform for behavior-based ecotoxicological studies, in combination with heart rate measurements, to be integrated into the *Daphnia magna* immobilization-based acute toxicity assay for enhancing its sensitivity to an ecotoxicity assessment of nano-sized PS NPs. This platform allowed the user-friendly and real-time integration of behavioral analysis with physiological outcome measurements during acute exposure of *Daphnia magna* to nano-sized carboxylated PS NPs. It enables real-time monitoring of living Daphnia movements and behavior in situ using a smartphone. In particular, the study addressed the effect of acute exposure of *Daphnia magna* to nano-sized carboxylated PS NPs on hearth rate and behavior, considering two variability factors, the age of the animals and the particle size. Neonate (aged less than 24 h) and adult (aged 7 days) were exposed to 40 nm and 200 nm carboxylated PS NPs, the last dimension generally being the biggest diameter for being called a NP in the biomaterials field.

## 2. Methods

### 2.1. Materials

A DaphtoxkitFM was purchased from MicroBioTest Inc., Gent, Belgium. A total of 200 nm (yellow-green, ex/em 505/515 nm) and 40 nm (dark red, ex/em 660/680) fluorescent carboxylate polystyrene particles were purchased from Thermofisher Scientific (Waltham, MA, USA). Mean hydrodynamic diameters and zeta potentials were estimated to be, respectively, 41 nm and −40 mV for 40 nm nanoparticles and 204 nm and −43 mV for 200 nm nanoparticles. Zeta potential of the PS nanoparticles was measured in ASTM hard water at pH ~8.0. Considering that *Daphnia magna* hemolymph pH ranges from 8.0 to 8.5 [30], no substantial change in zeta potential is expected upon internalization, indicating that the measured values are representative of in vivo conditions.

All chemicals were reagent-grade. Unless otherwise indicated, all chemicals were purchased from Sigma Aldrich (St. Louis, MO, USA).

### 2.2. Exposure of Neonates and Adults of Daphnia magna to Carboxylated PS Nanoparticles Under Acute Toxicity Test

The Daphtoxkit F™, which conforms with ISO Standard No. 6341:2012 [31] and OECD Guideline 202 [24], was used for the acute *Daphnia magna* test. This microbiotest based on the use of *Daphnia magna* specimens hatched from commercially available dormant eggs is a validated and widely used tool for conducting acute *Daphnia magna* tests. It offers a practical, cost-effective alternative to traditional methods based on the use of cultured organisms while maintaining high sensitivity and precision, making it suitable for both research and regulatory applications [32,33].

Standard freshwater, with a composition recommended by the ISO test protocols, was prepared from the concentrated salt solutions provided by the kit. The exposure experiment was carried out on the multiwall test plate provided by the kit. Ephippia were hatched after incubation for 72 h with a standard freshwater solution at 20 °C under continuous illumination of 6000 lux. Then neonates (aged less than 24 h) fed with a suspension of *Arthrospira platensis*, were divided into two groups: one was immediately exposed to PS nanoparticles while the other was cultured for a week at 20 °C and fed with *Arthrospira platensis*. The neonate group was divided in turn into two subgroups for the exposure experiment with 5 mg/L 200 nm and 40 nm carboxylated PS particles, respectively. In the same way, the adult specimens (non-pregnant specimens aged 7 days) were also divided in two subgroups and exposed to 5 mg/L 200 nm and 40 nm carboxylated PS nanoparticles, respectively. According to the kit protocol, 5 specimens were placed in each well containing a volume of 10 mL exposure medium. Each condition was repeated in four replicates.

Exposure was carried out for 48 h for both neonates and adults at 20 °C in the plates provided by the kit. Immobilization, assumed when the animal cannot swim within 15 s when gently prodded [24], was checked at 24 h and 48 h. The concentration used was 5 times higher than the highest possible concentration in environmental water [34], and it has already been used in other exposure experiments with *Daphnia* genus [35] in order to disclose the toxic effect and the underlying mechanisms of PS NPs under experimental laboratory conditions.

### 2.3. Heart Rate Effects

At the end of each assay, *Daphnia* specimens and one drop of exposure medium were carefully transferred using a clean pipette from the well plate onto a slide. The animal was observed under a light microscope equipped with computer-aided real-time imaging capability (IX 81 Inverted microscope, Olympus Corporation, Tokyo, Japan). Heart rate was assessed by video recording (60 s video) using a Nikon SMZ1270 stereomicroscope. The heartbeats were then manually counted from the one-minute recordings replayed in slow motion. Measurements were performed in three replicates on 4 specimens per condition.

### 2.4. Behavioral Effects Assessed by a Portable Smartphone-Based Platform

**Figure 1:**
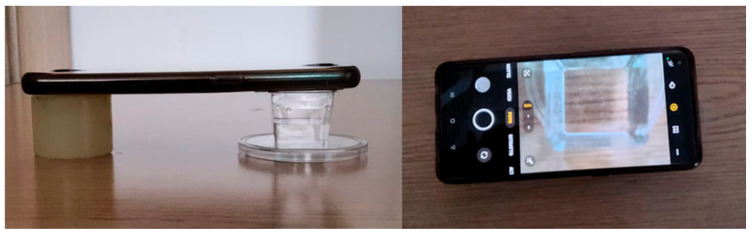
Smartphone-based tracking setup for behavioral analysis of *Daphnia magna*.

The behavior of *Daphnia* was investigated during the acute exposure experiment to PS NPs by means of video acquisitions integrated in the acute test using a smartphone camera mounted onto the multi-well test plate (Figure 1). This setup allowed behavioral analysis to be directly integrated within the acute toxicity exposure test. The smartphone platform enabled convenient monitoring of *Daphnia* movements and behavior through video recordings, facilitating observation of live individuals during the assay. This configuration permitted animal behavior to be recorded without transfer to an external optical bench or microscope stage, thereby avoiding alterations in locomotion that could arise from mechanical disturbance, light changes, or confinement during repositioning. In particular, swimming behavior of the daphnids was video-recorded for 1 min at 24 h (t24) and 48 h (t48) after application of the PS particles as well as in control groups at a frame rate of 60 fps. Video acquisition was performed using the smartphone’s built-in camera application. The videos were analyzed by a frame-by-frame method using the motion analysis software Tracker^®^, (version 6.1.6) from which two-dimensional trajectories ($x_{i}$, $y_{i}$) were extracted. To quantitatively describe locomotor activity, the trajectories were processed using Microsoft 365 Excel (version 2511) to calculate the total path length and the mean swimming speed [36,37]. The total path length L  was obtained as the sum of the consecutive Euclidean distances between sampled positions:

$$L=\sum_{i=1}^{N-1}\sqrt{{\left(x_{i+1}-x_{i}\right)}^{2}+{\left(y_{i+1}-y_{i}\right)}^{2}}$$

This measure represents the actual distance traveled by each individual during the recording period. The mean swimming speed was then computed as:

$$\bar{v}=\frac{L}{T}$$

where T is the effective duration of each video segment. This definition corresponds to the temporal average of the swimming velocity and avoids potential noise or numerical variability associated with instantaneous velocity estimates provided by the tracking software.

The x and y components of instantaneous velocity were computed from the tracked positions ($x_{i}$, $y_{i}$) as the frame-to-frame displacement divided by the time interval ∆t:

$$v_{x,i}=\frac{x_{i+1}-x_{i}}{∆t},\;v_{y,i}=\frac{y_{\;i+1}-y_{\;i}}{∆t}$$

The instantaneous velocity magnitude (modulus) was calculated as:

$$v_{i}=\sqrt{{v_{x,i}}^{2}+{v_{y,i}}^{2}}$$

Instantaneous velocities were also pooled across all individuals and represented as histograms by using Origin software 2016 to show the overall distribution of swimming speeds along the trajectory.

Instantaneous acceleration components were then calculated as the frame-to-frame changes in velocity components divided by the time separation between frames.

$$a_{x,i}=\frac{v_{x,i+1}-v_{x}}{∆t},\;a_{y,i}=\frac{v_{y,\;i+1}-v_{y}}{∆t}$$

The magnitude of instantaneous acceleration was obtained as:

$$a_{i}=\sqrt{{a_{x,i}}^{2}+{a_{y,i}}^{2}}$$

For each trajectory, the mean absolute acceleration $\bar{a}$ was extracted to describe overall locomotor activity. Measurements were performed on 10 specimens per condition.

### 2.5. Confocal Microscopy Observation

*Daphnia magna* specimens, both neonates and adults, either control or PS particle-exposed, were washed with standard freshwater and placed on a 35 mm glass-bottom dish with a drop of standard freshwater, reducing the surrounding liquid to a minimum to reduce movement. The distribution of PS particles in the animal body was determined using confocal laser scanning microscopy (Leica Microsystems, Mannheim, Germany). Excitation of yellow/green 200 nm fluorescent PS particles was carried out with an Ar laser (488 nm), while dark/red 40 nm fluorescent PS particles were excited with a HeNe laser (633 nm). Z-stack images with a 1 μm depth were collected. Both fluorescent and transmitted light images were recorded and overlayed to show both parameters in a single image.

### 2.6. Statistical Analysis

All the experiments were performed in triplicate. Statistical tests utilized to evaluate the statistical significance of differences were Student’s *t*-test, two-way ANOVA, and Tukey’s multiple comparison post-test as indicated in the figures’ captions.

## 3. Results

### 3.1. Effect of PS NPs on Daphnia magna Survival

First, the survival of *D. magna* neonates and adults exposed to 200 nm and 40 nm PS-NPs (5 mg/L) was assessed daily for a 48 h exposure. Both neonates and adults did not show any survival alterations to the exposure to either 200 nm or 40 nm particles for 24 or 48 h, the survival rate in all cases being higher than 98%.

### 3.2. Distribution of Carboxylated PS NPs in the Body of Daphnia magna Neonates and Adults

**Figure 2:**
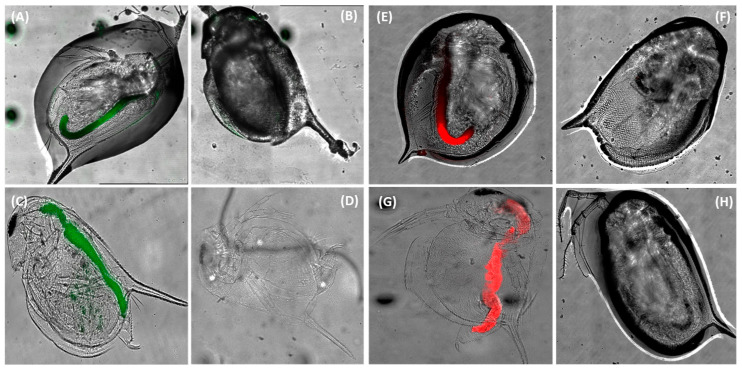
(**A**–**D**) Representative images of *Daphnia magna* exposed to 200 nm PS NPs (λ ex/em 505/515 nm, shown in green) for 48 h. (**A**) Adult treated animal; the accumulation in the gut is easily visible. (**B**) Adult control animal. (**C**) Neonate treated animal. (**D**) Neonate control animal. (**E**–**H**) Representative images of *D. magna* exposed to 40 nm PS NPs (λ ex/em 660/680 nm, shown in red) for 48 h. (**E**) Adult treated animal; the accumulation in the gut is easily visible. (**F**) Adult control animal. (**G**) Neonate treated animal. (**H**) Neonate control animal.

Then, the distribution of 200 nm and 40 nm carboxylated PS NPs in the body of neonates and adults of *D. magna* after 48 h exposure was detected by confocal microscopy. Figure 2A–D show the representative distribution of 200 nm PS NPs in an adult specimen (A) and in a neonate specimen (C) compared to the respective control animals (not exposed to the particles). The autofluorescence in the control specimens was negligible. The uptake of particles in the gut was clearly evident for both adults and neonates, where a slight fluorescence was also detectable at the thorax appendage level. The same pattern of distribution, with a prevalent accumulation in the gastrointestinal tract, was also observed for the 40 nm particles (Figure 2E–H). In this case, the autofluorescence of the control specimens was also negligible.

### 3.3. Effect of PS NPs on Daphnia magna Heart Rate

**Figure 3:**
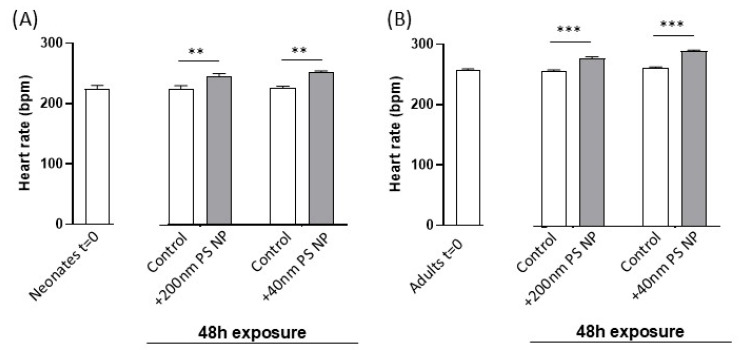
(**A**,**B**). Heart rate (measured as beats per min) in neonates (**A**) and adults (**B**) of *Daphnia magna* after 48 h exposure to 5 mg/L 200 nm PS NPs or 5 mg/L 40 nm PS NPs. Data are expressed as mean ± SD. ** *p* < 0.01; *** *p* < 0.001 (Student’s *t*-test).

The heart rate of neonates (A) and adults (B) after 48 h exposure to 40 nm and 200 nm PS NPs is reported in Figure 3. The graphs show the initial heart rate values for both neonates and adults and the values recorded following 48 h exposure. As observed by comparing the initial values of the heart rate of neonates and adults (Figure 3A,B), the mean neonate heart rate was slightly lower than the adult heart rate (224.3 ± 3.9 vs. 252.4 ± 5.1, *p* < 0.05, Student’s *t*-test).

Following exposure to PS NPs, a significant mean increment of 26 and 20 beats per min was observed in neonates exposed to 40 nm and 200 nm, respectively (about 1.11 and 1.09 times higher compared to control group). Similar results were obtained for adults, with a significant increment of 29 and 21 beats per minute following exposure to 40 nm and 200 nm PS NPs, respectively (about 1.11 and 1.08 times higher compared to control). Then, we performed two-way Analysis of Variance including the two variability sources “age” and “PS NP exposure”, applied to the 48 h data set. Two-way ANOVA confirmed a significant (*p* < 0.001) influence of age on the heart rate, since it was lower in neonates on average as already observed, and confirmed a significant (*p* < 0.001) effect of the exposure to PS NPs. No significant interaction between the two variability factors was registered, suggesting that the effect of the exposure to PS NPs, either 40 nm or 200 nm, on heart rate did not change with the age of the animals.

### 3.4. Effect of PS NPs on Daphnia magna Behavior

**Figure 4:**
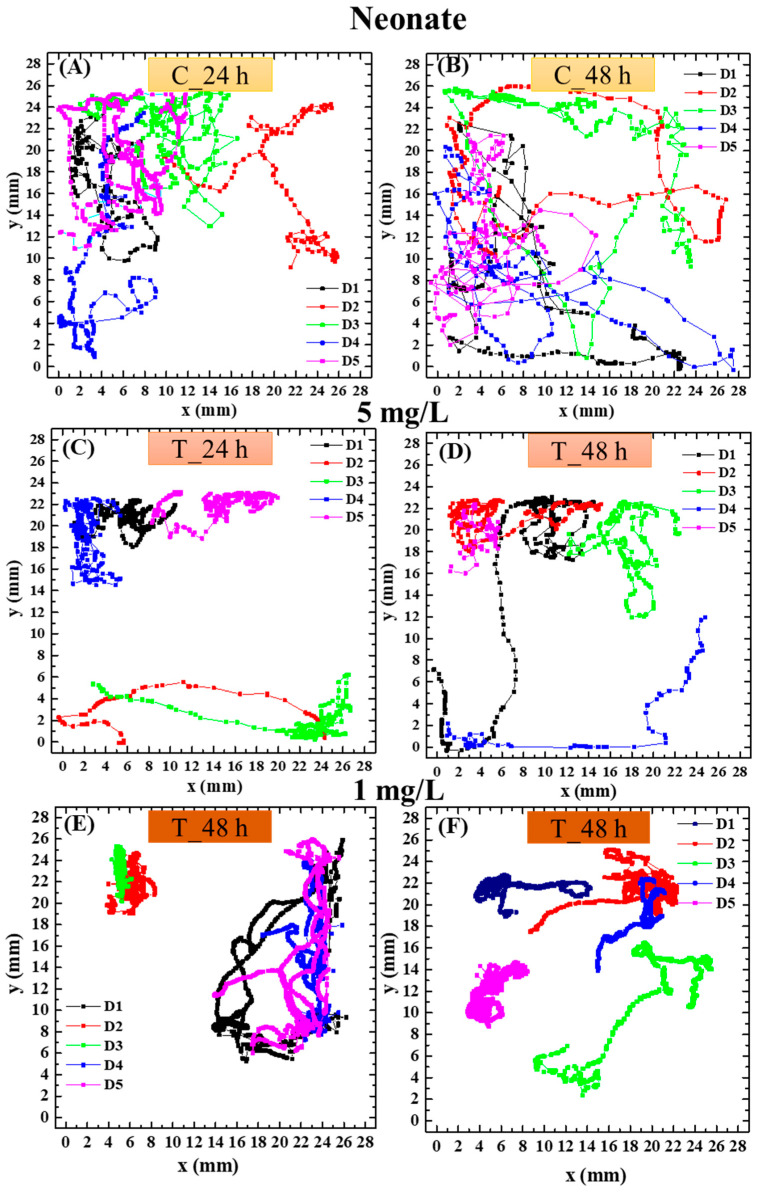
(**A**,**B**) Swimming trajectories of nontreated neonate *Daphnia magna* and after 24 h (**C**,**E**) and 48 h (**D**,**F**) of exposure to 40 nm PS NPs at a concentration of 5 mg/L (**C**,**D**) and 1 mg/L (**E**,**F**).

**Figure 5:**
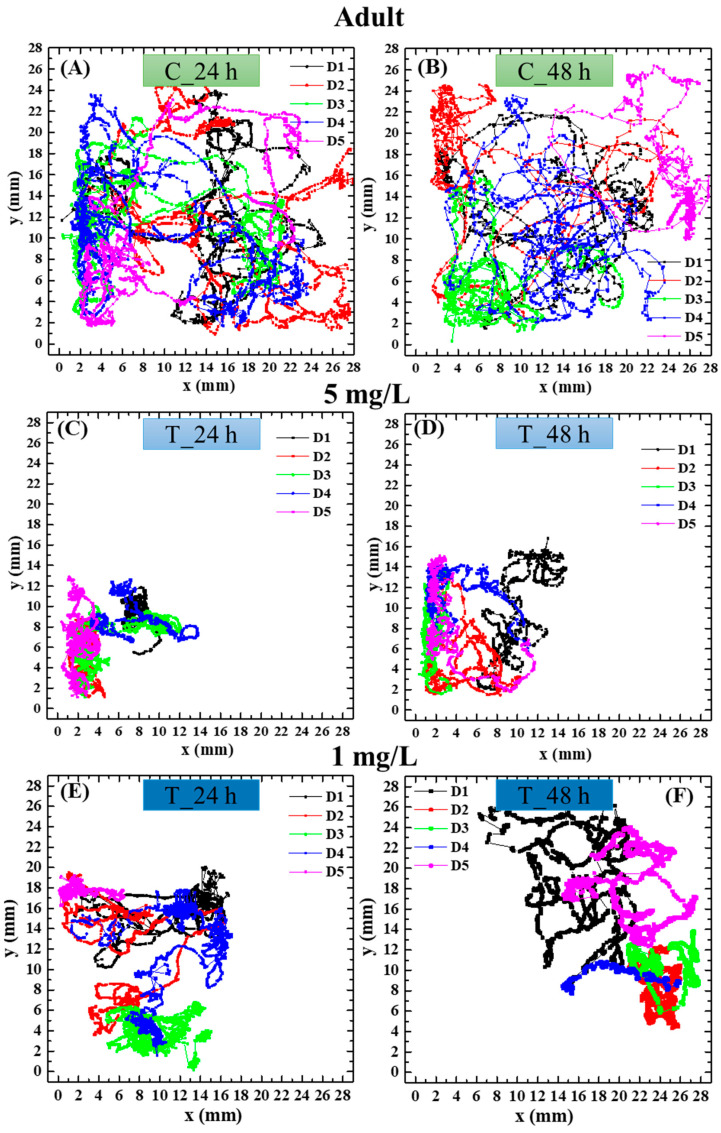
(**A**,**B**) Swimming trajectories of nontreated adult *Daphnia magna* and after 24 h (**C**,**E**) and 48 h (**D**,**F**) of exposure to 40 nm PS NPs at a concentration of 5 mg/L (**C**,**D**) and 1 mg/L (**E**,**F**).

Exemplary swimming trajectories of neonate and adult daphnids of the control group and those treated with polystyrene nanoparticles of 40 nm diameter at t24 and t48 are shown, respectively, in Figure 4 and Figure 5. To enhance the clarity of data visualization, only 5 of the 10 analyzed replicates are displayed in the figure as representative examples. It was observed that trajectories of the treated daphnids are different from those of the nontreated ones. Notably, control animals move almost across the whole observation area, while daphnids exposed to polystyrene nanoparticles of 40 nm swim mainly around small areas close to the walls. This effect was not significant with nanoparticles of 200 nm.

**Figure 6:**
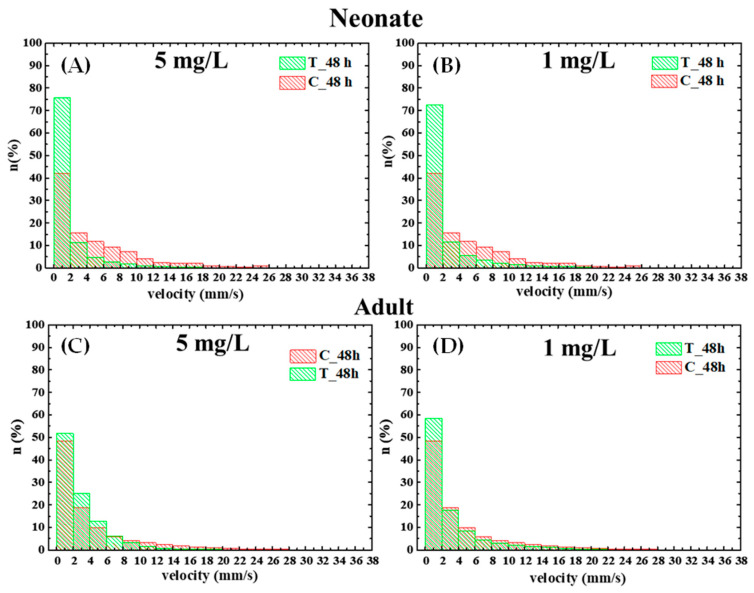
(**A**,**B**) Histogram of swimming velocities of nontreated neonate and (**C**,**D**) adult *Daphnia magna* and after 48 h exposure to 40 nm PS NPs at different concentrations (5 mg/L and 1 mg/L).

Moreover, as shown in Figure 6, exposure to PS nanoparticles for 48 h led to a dose-dependent reduction in total path length, mean swimming velocity, and acceleration in both neonate and adult daphnids. In neonates, the total path length decreased from 293 ± 10 mm in controls to 219 ± 15 mm and 197 ± 7 mm at 1 mg/L and 5 mg/L, respectively, with a corresponding decrease in mean velocity from 4.9 ± 0.2 mm/s to 3.7 ± 0.3 mm/s and 3.3 ± 0.2 mm/s and in mean acceleration from 340 ± 7 mm/s^2^ to 254 ± 20 mm/s^2^ and 220 ± 7 mm/s^2^. In adults, the total path length decreased from 332 ± 40 mm in controls to 240 ± 30 mm and 216 ± 15 mm at 1 mg/L and 5 mg/L, respectively, with mean velocity dropping from 5.5 ± 0.7 mm/s in controls to 3.9 ± 0.5 mm/s at 1 mg/L and 3.6 ± 0.3 mm/s at 5 mg/L and mean acceleration from 300 ± 20 mm/s^2^ to 270 ± 40 mm/s^2^ at 1 mg/L and 250 ± 20 mm/s^2^ at 1 mg/L. Analysis of instantaneous velocities from the histograms, as shown in Figure 6, further confirmed a decrease in swimming activity at both concentrations.

## 4. Discussion

The development of more sensitive and user-friendly bioassays is crucial for improving micro- and nanoplastic ecotoxicity assessments and enhancing the comprehension of the mechanisms underlying their toxicity. Although nanoplastics represent an environmental and public health concern, the mechanisms of their toxicity on living organisms are poorly understood to date. The integration of novel sensitive assays into routine testing can enhance the detection of subtle toxic effects and improve the relevance of toxicity assessments. In the present work a portable smartphone-based platform for behavior-based ecotoxicological studies was integrated into the *Daphnia magna* immobilization-based acute toxicity assay in combination with heart rate measurements for enhancing the sensitivity of the traditional test to an ecotoxicity assessment of nano-sized PS NPs. The study considered three variability factors that could influence the biological effects of NPs: the size and concentration of the particles and the age of the organisms.

Daphnid physiological attributes such as heart rate and behavior are considered sensitive endpoints useful for rapid screening of the toxicity of environmental aqueous samples, drugs, and contaminants [26]. Heart rate represents a useful indicator of the physiological status of the organism, being very sensitive to a number of stressful conditions [37,38,39]. The heart of *Daphnia* sp. is clearly visible through the transparent carapace and its contraction rate can be easily monitored in a non-invasive way. The *Daphnia* heart has been widely used as a model organ for the study of the cardiotoxic effects of pharmaceuticals [40], but it has been utilized little for the assessment of the effects of nanoparticles [26]. *D. magna* has a myogenic heart, like mammalian hearts, and it is also known to be susceptible to cardioactive drugs known to affect the human heart [40]. All these advantages make *D. magna* an excellent animal model to investigate the effect of environmental contaminants on the cardiovascular system. Behavior is also considered an early and sensitive indicator of toxicity in several species [41]. In particular, in *Daphnia*, changes in swimming behaviors have been shown to affect predation risk [42] and, in turn, animal survival [39,43]. Moreover, several pieces of experimental evidence demonstrated the sensitivity of *Daphnia* swimming behavior to pesticides, nanoparticles, bacterial products, and several chemicals. The analysis of behavioral aspects is recognized as a novel tool for water-quality monitoring and assessment [37].

In the present work we first demonstrated that carboxylated PS nanoparticles, either 200 nm and 40 nm, do not affect survival in the studied organisms after 48 h exposure at the concentration used, 5 mg/L, and then we assessed their body distribution. The presence of both 200 and 40 nm PS NPs in the alimentary canal of the animals after 48 h exposure was clearly evident, while the particles were not clearly detectable in other body compartments. Cui et al. [35] recorded the accumulation of uncharged 50 nm PS NPs, at the same concentration as in the present study, from the external surface of the body to the internal organ of adult *Daphnia galeata* after 5 days’ exposure. Our results compared to the literature data could be explained by the shorter exposure period and/or the negative charge of the PS NPs, which could prevent the observation of detectable NP accumulation in other body compartments.

Although no significant effects were detected on survival within the short 48 h timeframe of the acute test, a statistically significant increase in the heart rate of both adults and neonates after 48 h exposure to carboxylated PS NPs was observed. The effects were detected for both 40 nm and 200 nm particles and, did not change with the age of the animals. As recently outlined by [44], the study of the effects of micro/nanoplastics on the cardiovascular system of organisms is of particular importance due to the the system’s high sensitivity to environmental changes and its role in transporting substances to other organ systems. The effects and the underlying mechanisms of nanoplastics on cardiocirculatory systems are to date largely unknown. Most of the information about nanoplastics on heart functioning arises from toxicological studies on zebrafish. As recently reviewed by Torres-Ruiz [45], zebrafish larvae heart rate alterations were observed in over half of the articles reviewed, but no correlation was found with particle size/concentration or exposure time. Bradycardia was the most common effect observed but tachycardia was also recorded in some cases. One study (Duan et al., 2020 [46]) detected both effects depending on the exposure time (tachycardia at 24 h and bradycardia at 48 h). The *Daphnia* heart is composed of a thin layer of myocardial cells, making it easily modulated by compound agents present in the medium in which the organisms swim. The interaction between negatively charged nanoparticles and cardiac tissue has been studied in vitro on rat myocytes [47]. These studies demonstrated that 50 nm carboxyl-modified polystyrene latex nanoparticles triggered the formation of nanopores in the membrane, leading to pro-arrhythmic events, reduced conduction velocity, and a pathological increase in action potential duration, together with an increase in ionic current throughout the membrane [47]. Stampfl et al. [48] observed an increase in heart rate in a mammalian model of ex vivo isolated heart exposed to a nanoparticle suspension and attributed the observed effects to the increased release of catecholamines from the neural endings within the heart. In our model system a possible explanation of the tachycardic effect observed in both neonates and adults following exposure to both 40 nm and 200 nm PS NPs could be attributed to an interference of carboxylated PS NPs with adrenergic-type receptors, exerting an agonistic effect.

As regards behavioral responses of PS NP exposure, they were analyzed by the portable smartphone-based platform developed in this study, which allowed the real-time integration and monitoring of behavioral analysis of the animals during acute exposure experiments. Although conventional optical benches offer high optical performance, their use typically requires removing organisms from the exposure plate and positioning them on a microscope stage. These steps can introduce stress that can alter behavioral endpoints, potentially confounding the results. In contrast, our smartphone-based setup enabled continuous, non-invasive monitoring of freely swimming *Daphnia* directly within the acute toxicity setup, preserved animal behavior, and reduced manipulation-induced artifacts. Quantitatively, the smartphone-based setup provided an adequate spatial resolution and frame rate (60 fps) for reliable *Daphnia* motility tracking, as confirmed by the consistency of the velocity, acceleration, and trajectory parameters obtained. Moreover, in addition to laboratory use, the compact format of the smartphone-based platform provides a viable basis for on-field short-term ecotoxicity assays. Its ability to acquire behavioral data directly within exposure wells eliminates the need for stationary optical instrumentation and enables rapid testing at the site of sample collection. Furthermore, the native digital output of the system is readily compatible with IoT cloud architectures, allowing automated upload, remote processing, and standardized behavioral analytics across monitoring locations. In addition, although the selected contaminant (carboxylated PS nanoparticles) induces relatively slow behavioral alterations—making immediate in-field deployment less critical—the advantage of a portable platform becomes much more relevant in the presence of pollutants with a rapid onset of action, where timely ecotoxicological response is essential. By validating the platform under slower and more challenging exposure conditions, we adopted a conservative scenario: if the system reliably detects subtle behavioral changes that develop gradually, its performance is expected to be equal or superior when applied to contaminants that elicit acute and fast behavioral disruption.

The portable smartphone-based platform permitted the detection of subtle effects such as alterations in trajectories, path length, mean velocity, and acceleration. This allowed the expansion of the biosensing potential of the traditional acute test, thereby increasing sensitivity to subtle effects of nanoplastics that may occur even when mortality is unaffected within the short exposure window of the OECD test. In particular, significant behavioral changes were observed in both neonates and adults exposed for 48 h to 40 nm particles compared with the control specimens, but no significant effect was observed on animals exposed to 200 nm particles. Velocity is one of the most widely used descriptors of *Daphnia* behavioral activity. Mean velocity is a key indicator of microcrustacean activity levels, as it directly reflects how actively these organisms are swimming or moving in their environment. Changes in mean velocity can signal behavioral responses to environmental stressors [49]. Acceleration is a parameter measured in the analysis of *Daphnia* behavior, particularly when examining swimming patterns. Acceleration refers to the rate of change in velocity, indicating rapid bursts in animal motility [50].

In the literature, the exposure to several types of nanoparticles, such as titanium dioxide, cerium dioxide, multi-wall carbon nanotubes, graphene, graphene oxide, and carbon fullerene C, has been demonstrated to decrease swimming speed in a concentration-dependent manner [38,51,52,53,54]. In our work 40 nm carboxylated PS NPs shared the ability to decrease the swimming velocity with other nanomaterials. Possible underlying mechanisms suggested to explain the size-dependent effects of nanomaterials on the swimming velocity of *Daphnia* are related to the possible induced oxidative stress and/or the physical interferences of the nanoparticles with the daphnids’ carapace [55]. In the case of carboxylated PS NPs in our work, the effect appears to be size-dependent, since 200 nm particles did not exert any significant effect.

Moreover, trajectories appeared altered in 40 nm PS NP-exposed organisms compared to control organisms. Control animals showed less curved trajectories which covered a wide area of the observation dish, while the tracks of the animals treated with 40 nm PS NPs developed only around small areas close to the walls, showing a possible loss of orientation. Both age groups showed similar effects of 40 nm polystyrene nanoplastics on their behavior, with comparable patterns of alteration in trajectories characterized by increased spatial confinement of the animals’ movements compared to the control organisms and a reduction in speed in both age classes after 48 h of exposure, slightly more pronounced in adults compared to neonates. A slight concentration-dependent trend was also detected for the 40 nm PS NPs, with the impairment in swimming parameters being moderately more marked at 5 mg/L than at 1 mg/L. Reductions in swimming velocity, path length, and acceleration, together with altered trajectories in daphnids exposed to 40 nm carboxylated PS NPs, indicate a neurofunctional effect of these nanoparticles, manifested through measurable behavioral changes.

By integrating the results of behavior and heart rate, we observed an increase in heart rate and a decrease in velocity, along with altered trajectories for the smaller 40 nm particles. A similar pattern of responses has been previously demonstrated for *Daphnia* exposed to low concentrations of chemicals [56,57] as well as for other fish model species such as zebrafish and medaka, which were exposed to pesticides and analyzed using the DanioVision Observation Chamber (DVOC) [58].

## 5. Conclusions

In conclusion, obtained results demonstrated the suitability of the proposed smartphone platform, enabling user-friendly and real-time integration of behavioral analysis with physiological outcome measurements during acute exposure of *Daphnia magna* to nano-sized carboxylated PS NPs, expanding the sensitivity of the traditional acute toxicity tests. This approach integrates physiological and behavioral endpoints for a more comprehensive assessment of nanoplastic toxicity.

By applying this integrated approach, it was possible to detect a significant impact of PS carboxylated nanoparticles on the heart rate and behavior of *Daphnia magna* under short-term (48 h) exposure. In particular, stimulation of the heart rate was observed for both neonates and adults with the two particle sizes, 40 nm and 200 nm, presumably attributable to the interference of carboxylated PS NPs with adrenergic-type receptors, exerting an agonistic effect. The impact of nanoparticle exposure on behavior was detectable for 40 nm particles but not for 200 nm ones and consisted of a decrease in velocity, acceleration, and alterations in trajectories. These changes suggest that these particles impair orientation and movement efficiency, highlighting their neurotoxic potential. While both particle sizes accumulated primarily in the gastrointestinal tract, only the smaller 40 nm particles led to noticeable behavioral impacts. This underscores the importance of nanoparticle size in determining toxicological outcomes.

The study highlights the need for sensitive, sub-lethal endpoints (e.g., heart rate and swimming behavior) in routine toxicity tests to detect early and subtle toxic effects, especially for emerging pollutants like nanoplastics. In this regard, the integration of behavioral analyses through a user-friendly, smartphone-based platform offers a novel, cost-effective, and field-applicable method for environmental monitoring of nanoparticle toxicity. The use of such miniaturized, portable smartphone platforms can also enable on-field short-term ecotoxicity tests with IoT cloud support for behavioral analysis.

## Data Availability

Data supporting reported results are statistically discussed in the manuscript. Further inquiries can be directed to the corresponding authors.
